# Extracellular membrane vesicles and nanotubes in Archaea

**DOI:** 10.1093/femsml/uqab007

**Published:** 2021-06-10

**Authors:** Junfeng Liu, Nicolas Soler, Aurore Gorlas, Virginija Cvirkaite-Krupovic, Mart Krupovic, Patrick Forterre

**Affiliations:** Archaeal Virology Unit, Institut Pasteur, 75015 Paris, France; Université de Lorraine, INRAE, DynAMic, F-54000 Nancy, France; Université Paris-Saclay, CEA, CNRS, Institute for Integrative Biology of the Cell (I2BC), 91198 Gif-sur-Yvette, France; Archaeal Virology Unit, Institut Pasteur, 75015 Paris, France; Archaeal Virology Unit, Institut Pasteur, 75015 Paris, France; Université Paris-Saclay, CEA, CNRS, Institute for Integrative Biology of the Cell (I2BC), 91198 Gif-sur-Yvette, France; Département de Microbiologie, Institut Pasteur, 75015 Paris, France

**Keywords:** extracellular vesicles, nanotubes, archaea, vesiduction, extremophiles, DNA transfer

## Abstract

Membrane-bound extracellular vesicles (EVs) are secreted by cells from all three domains of life and their implication in various biological processes is increasingly recognized. In this review, we summarize the current knowledge on archaeal EVs and nanotubes, and emphasize their biological significance. In archaea, the EVs and nanotubes have been largely studied in representative species from the phyla Crenarchaeota and Euryarchaeota. The archaeal EVs have been linked to several physiological processes such as detoxification, biomineralization and transport of biological molecules, including chromosomal, viral or plasmid DNA, thereby taking part in genome evolution and adaptation through horizontal gene transfer. The biological significance of archaeal nanotubes is yet to be demonstrated, although they could participate in EV biogenesis or exchange of cellular contents. We also discuss the biological mechanisms leading to EV/nanotube biogenesis in Archaea. It has been recently demonstrated that, similar to eukaryotes, EV budding in crenarchaea depends on the ESCRT machinery, whereas the mechanism of EV budding in euryarchaeal lineages, which lack the ESCRT-III homologues, remains unknown.

## Teaser

Extracellular vesicles and nanotubes could play a profound role in archaeal physiology, evolution and environmental adaptation by promoting intercellular transfer of viral and cellular nucleic acids and proteins.

## INTRODUCTION

Archaea have been recognized as a separate domain of life, besides Bacteria and Eukarya, only in 1977 by Carl Woese and colleagues who compared the ribosomal RNA (rRNA) gene sequences from diverse organisms (Woese and Fox 1977). The name Archaea, instead of archaebacteria, was proposed later on by Carl Woese to emphasize the fact that Archaea and Bacteria form two distinct lineages in the universal tree of life (Woese, Kandler and Wheelis 1990). Although archaeal cells are of the prokaryotic type, their informational systems (DNA replication, transcription, translation), as well as several membrane-associated machineries, such as the ATP synthase complex, the Sec secretion system and the signal recognition particles, are much more similar to those of eukaryotes. Similar to bacteria and eukaryotes, archaeal cells commonly secrete extracellular vesicles (EVs) and some produce tubular structures resembling bacterial nanopods and/or nanotubes (Gill, Catchpole and Forterre 2019).

The production of various types of EVs (apoptotic bodies, exosomes, microvesicles, etc.) and nanotube-like structures (tunnelling nanotubes, etc.) has been extensively studied in Eukarya (for reviews, see Yanez-Mo *et al*. 2015; Gill, Catchpole and Forterre 2019; Cordero Cervantes and Zurzolo 2021). In Bacteria, EVs were observed for the first time by electron microscopy in *E. coli* in 1966 (Knox, Vesk and Work 1966), but their biological importance was first dismissed and, hence, microbial EVs have been considered as artefacts of cell growth or lysis for many years (Coelho and Casadevall 2019). However, bacterial EVs are increasingly recognized to play important roles in many processes from pathogenesis, bacterial communication and biofilm formation, to horizontal gene transfer and protection against viral infections (Brown *et al*. 2015; Jan 2017; Gill, Catchpole and Forterre 2019). Nanotubes have been described in Bacteria more recently (Baidya *et al*. 2018; Gill, Catchpole and Forterre 2019), but their physiological role remains controversial (Baidya, Rosenshine and Ben-Yehuda 2020; Pospíšil *et al*. 2020). In Archaea, EVs have been first described over two decades ago when they were found to carry protein toxins (Prangishvili *et al*. 2000). Although until recently, archaeal EVs received relatively little attention, perhaps due to the fact that their discovery was from the very beginning linked to a defined function (i.e. toxin transfer), archaeal EVs were not dismissed by the archaeal community as cellular ‘junk,’ as in some other branches of microbiology (Coelho and Casadevall 2019). The research on archaeal EVs has primarily focused on two orders of hyperthermophilic species, Sulfolobales and Thermococcales, whereas nanotubes have been primarily described in Thermococcales and Haloarchaea.

A major difference between Archaea and Bacteria that probably influences the respective mechanisms of EV and nanotube production is the structure of their cell envelopes. Similar to most eukaryotic cells, most Archaea are monoderms, i.e. their envelope consists of a single membrane (Ellen *et al*. 2010; Albers and Meyer 2011). Important exceptions are *Ignicoccus hospitalis* and members of the order Methanomassilicoccales that are diderm archaea with an outer membrane and a periplasmic space (Dridi *et al*. 2012; Klingl 2014; Heimerl *et al*. 2017). Most archaea lack rigid cell wall and this may facilitate the production of EVs and nanotubes. The exceptions are methanogenic archaea of the orders Methanobacteriales and Methanopyrales in which the membrane is surrounded by a peptidoglycan-like polymer, referred to as pseudomurein layer (Steenbakkers *et al*. 2006; Albers and Meyer 2011; Klingl 2014), and halophilic archaea of the genus *Halococcus* that are surrounded by a complex and rigid heteroglycan cell wall (Steber and Schleifer 1975). The cytoplasmic membrane is surrounded by a paracrystalline protein surface (S-) layer usually composed of a single main glycoprotein (40–200 kDa) that is capable of self-assembly into highly ordered structures (Rodrigues-Oliveira *et al*. 2017). As in eukaryotes, the outer surfaces of archaea are also often covered with abundant glycoproteins and the protein N-glycosylation pathways exhibit important similarities between Archaea and Eukarya, suggesting a common origin (Nikolayev, Cohen-Rosenzweig and Eichler 2020).

For a long time, Archaea have been divided into two major phyla based on rRNA sequence comparisons: Crenarchaeota and Euryarchaeota (Woese, Kandler and Wheelis 1990). Crenarchaeota includes thermophilic or hyperthermophilic species, whereas members of Euryarchaeota are phenotypically very diverse, including (hyper)thermophiles, mesophiles, methanogens and halophiles. Most cultivated species belong to these two phyla and all experimental studies on archaeal EVs have been performed either on Crenarchaeota (order Sulfolobales) or Euryarchaeota (order Thermococcales and class Halobacteria). In 2008, a third major archaeal phylum was proposed, the Thaumarchaeota (Brochier-Armanet *et al*. 2008). Many thaumarchaeal species, either mesophilic or thermophilic, have now been cultivated, but they all turned out to be very fastidious and the production of EVs has not yet been studied in this phylum.

Although many eukaryotic features are common to all archaea, others are specific to only some lineages, phyla or superphyla. This is the case for the endosomal sorting complexes required for transport (ESCRT) machinery proteins that are responsible not only for cell division but also for EV biogenesis in eukaryotes (Vietri, Radulovic and Stenmark 2020). Both eukaryotic-like proteins of the ESCRT machinery, ESCRT-III and Vps4 ATPase, are conserved in Crenarchaeota (except for Thermoproteales). Members of the Thaumarchaeota also encode the ESCRT proteins that are very similar to those of Crenarchaeota (Caspi and Dekker 2018; Lu *et al*. 2020). However, despite producing EVs, members of the order Euryarchaeota, such as Thermococcales and Halobacteriales, do not encode the ESCRT machinery but only Vps4 homologues. Instead, the euryarchaeal lineage relies on the bacterial-like FtsZ-based system for cell division. This suggests that EV biogenesis in different archaeal lineages occurs by very different mechanisms. Thus, mechanistic comparisons of EV budding in archaea and eukaryotes might provide insights into the evolution and diversification of this important process in different cellular lineages.

In recent years, a wealth of new archaeal phyla have been described from the reconstructions of metagenome-assembled genomes, vastly expanding the known diversity of Archaea and the range of their ecological distribution (Adam *et al*. 2017; Spang, Caceres and Ettema 2017). Several new phyla corresponding to small archaea have been grouped in the DPANN superphylum (referring to the first described constituent lineages, Diapherotrites, Parvarchaeota, Aenigmarchaeota, Nanoarchaeota and Nanohaloarchaeota) (Rinke *et al*. 2013). ESCRT machinery proteins and many other eukaryotic-like proteins missing in Euryarchaeota are also absent in DPANN archaea. These miniature archaea have reduced genomes and most of them lack essential metabolic pathways, such as lipid biosynthesis, suggestive of parasitic lifestyles. Consequently, most DPANN members are probably ectosymbionts of larger archaeal hosts (Dombrowski *et al*. 2019). The best characterized of these symbiotic interactions is the association of *Nanoarchaeum equitans* (a member of the Nanoarchaeota) with its host, the diderm crenarchaeon *Ignicoccus hospitalis*. Remarkably, membrane vesicles that might be implicated in the interactions between *Nanoarchaeum* and *Ignicoccus* have been observed budding from the internal cytoplasmic membrane of *I. hospitalis* (see later) (Junglas *et al*. 2008).

Another major superphylum, the Asgardarchaeota, has attracted much attention because some phylogenetic analyses of universal protein concatenation have suggested that eukaryotes emerged from within this phylum (Spang *et al*. 2015; Zaremba-Niedzwiedzka *et al*. 2017; Williams *et al*. 2020). This hypothesis is still debated because Archaea are monophyletic, with Asgard archaea branching between euryarchaea and crenarchaea in other universal protein phylogenies (Da Cunha *et al*. 2017, 2018; Jay *et al*. 2018). Interestingly, the genomes of Asgardarchaeota encode more eukaryotic-like proteins than most other archaeal phyla (Zaremba-Niedzwiedzka *et al*. 2017), including actin, tubulin and additional components of the ESCRT system (Caspi and Dekker 2018; Lu *et al*. 2020). Moreover, these proteins are also more closely related to their eukaryotic homologues than those from other Archaea. Asgard archaea should therefore serve as valuable models to study the role of these proteins in the biogenesis of archaeal EVs and/or nanotubes. However, only one Asgard archaeon, *Candidatus* Prometheoarchaeum syntrophicum, has been successfully cultivated yet, and only in symbiosis with other microorganisms (Imachi *et al*. 2020). Interestingly, electron microscopy analyses have suggested that this organism produces both EVs and nanotubes (see later).

## PRODUCTION OF EVS BY ARCHAEA OF THE PHYLUM CRENARCHAEOTA

Nearly all studies dealing with the production of EVs in crenarchaea have been performed in organisms of the order Sulfolobales. These archaea are thermoacidophiles thriving in acidic (pH 2–3) terrestrial hot springs that have been studied as model organisms by biochemists and molecular biologists from the very beginning of archaeal research, mainly because they are aerobes and are easy to cultivate (Schocke, Brasen and Siebers 2019). Moreover, many genetic tools are now available for several *Sulfolobus* species (Peng *et al*. 2017). The earliest reports about archaeal EVs came from studies carried out on *Sulfolobus islandicus* (Prangishvili *et al*. 2000). These EVs, 90–230 nm in diameter and coated with the S-layer, were shown to be associated with an antimicrobial protein, termed ‘sulfolobicin,’ that inhibits the growth of related *Sulfolobus* species. Subsequently, EV-associated toxins were identified in some other *Sulfolobus* species and were shown to be encoded by a two-gene operon (Ellen *et al*. 2011). Disruption of these genes has shown that the two sulfolobicin proteins, dubbed SulA and SulB, are required for the antimicrobial activity (Ellen *et al*. 2011). Notably, their association with EVs was not necessary for the antimicrobial activity since purified sulfolobicins were still active once extracted from EVs by alkaline carbonate treatment.

Characterization of EVs produced by *Sulfolobus acidocaldarius*, *Saccharolobus solfataricus* and *Sulfurisphaera tokodaii* showed that lipid and protein profiles of the parental cells were different from those of the corresponding EVs (Ellen *et al*. 2009). Interestingly, the protein content analyses have shown that EVs from all three species carry some components of the ESCRT machinery, namely, ESCRT-III-1 and ESCRT-III-2 and the Vps4 ATPase (also called CdvC) (Ellen *et al*. 2009). All three proteins play a key role in the *Sulfolobus* cell division (Lindås *et al*. 2008; Samson *et al*. 2008; Liu *et al*. 2017; Tarrason Risa *et al*. 2020). Given that in eukaryotes the ESCRT machinery is responsible not only for the cytokinesis but also among other functions, for EV budding (Vietri, Radulovic and Stenmark 2020), it has been hypothesized that production of *Sulfolobus* EVs also depends on the ESCRT machinery.

**Figure 1.**
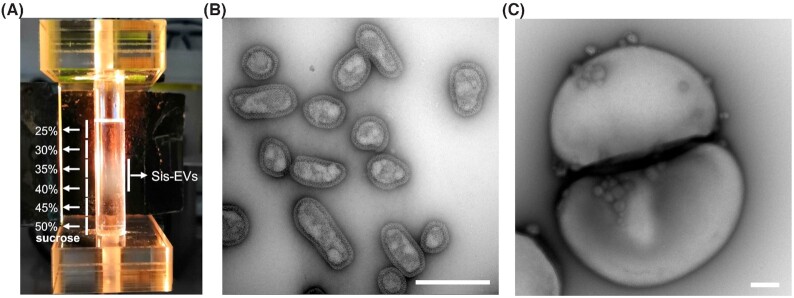
Extracellular vesicles produced by *Sulfolobus islandicus*. **(A)** EV purification by ultracentrifugation in the 25–50% sucrose gradient. EVs form an opalescent band in the region corresponding to 30–40% sucrose. The figure is modified from Liu *et al*. (2021b). **(B)**Transmission electron micrograph of negatively stained EVs. **(C)** EVs budding from a dividing *Sulfolobus* cell. Scale bars: 400 nm.

Direct evidence that budding of *Sulfolobus* EVs depends on the ESCRT machinery has been recently provided by an in-depth characterization of the EVs from *S. islandicus* (Liu *et al*. 2021b). Proteomic analysis has shown that highly purified *S. islandicus* EVs (Sis-EVs; Fig. 1A and B) carry 413 proteins, including all six components of the *Sulfolobus* ESCRT machinery, with ESCRT-III-2 and ESCRT-III-1 being in the top-10 of the most abundant EV proteins. Western blot analysis confirmed that both proteins were present and strongly enriched in the Sis-EVs. Using a CRISPR-based knockdown system, it was demonstrated that the four archaeal ESCRT-III homologues and the AAA+ ATPase Vps4 were all required for EV production, whereas the archaea-specific component CdvA appeared to be dispensable (Liu *et al*. 2021b). Importantly, using synchronized *S. islandicus* cultures, it was shown that EV production is linked to cell division (Fig. 1C) and coincides with the natural, cell cycle-linked changes in the expression of ESCRT-III homologues, in particular, ESCRT-III-1 and ESCRT-III-2. Consistently, overexpression of ESCRT-III-1 and ESCRT-III-2 from a plasmid resulted in 200–250% increase in vesiculation, while overexpression of other ESCRT machinery components had little effect on EV production (Liu *et al*. 2021b). Based on these findings, it has been suggested that ESCRT-mediated EV biogenesis has deep evolutionary roots and predates the divergence of eukaryotes and archaea. Interestingly, it has been recently shown that in virus-infected *S. islandicus* cells, ESCRT machinery mediates asymmetric cell division, whereby normal-sized cells are budding from the giant virus-infected cells (Liu *et al*. 2021a), topologically resembling the EV budding.

A hypervesiculation phenotype was obtained in cells overexpressing the CdvA protein (Liu *et al*. 2021b). The latter protein does not appear to participate in normal EV budding, because CRISPR-mediated depletion of the *cdvA* transcripts had little effect on the EV production. However, when the CdvA was overexpressed from a plasmid, the EV production was boosted by several folds. It was suggested that the hypervesiculation is the result of excessive binding of CdvA to the membrane (Liu *et al*. 2021b). Regardless of the exact mechanism, it appears that EVs can be produced by different mechanisms even in the same organism. The normal EV production is evidently linked to cell division in *Sulfolobus* but it is possible that under different physiological conditions and upon exposure to different stressors EVs could be produced through different pathways.

Besides ESCRT machinery components, Sis-EVs carry a diverse subset of the *S. islandicus* proteome, including diverse proteases and nucleases (Liu *et al*. 2021b). However, highly purified Sis-EVs showed no toxicity against other *Sulfolobus*, *Saccharolobus* and *Sulfurisphaera* species tested, suggesting that EV-mediated transfer of sulfolobicins might not be a general phenomenon. Notably, proteins carried by Sis-EVs were not randomly included from the *S. islandicus* proteome. Indeed, comparison of the Sis-EV and *S. islandicus* proteomes showed that Sis-EV protein fraction is strongly enriched in membrane proteins as well as proteins from particular functional categories, including the cell division (as discussed earlier), cell motility, posttranslational modification, protein turnover and secretion as well as energy production and conversion, and inorganic ion transport and metabolism (Liu *et al*. 2021b).

Sis-EVs cargo includes not only diverse proteins but also chromosomal and plasmid DNA. Importantly, Sis-EVs protect the cargo DNA from nucleases as well as the harsh physicochemical conditions of the extracellular milieu and can transfer it to recipient cells (Liu *et al*. 2021b). The possibility to transfer DNA via EVs was previously observed with archaea of the order Thermococcales (see later) and also in bacteria (Domingues and Nielsen 2017). The term ‘vesiduction’ has been proposed for DNA transfer mediated by EVs as a fourth way of DNA transfer, besides transformation, transfection and conjugation (Soler and Forterre 2020).

Moreover, Sis-EVs can also support the heterotrophic growth of *S. islandicus* in minimal medium, implicating EVs in carbon and nitrogen fluxes in extreme environments. Thus, it is becoming clear that EVs play an important role in horizontal gene transfer and nutrient cycling in extreme environments. Indeed, S-layer-covered EVs have been detected directly in an environmental sample collected from a terrestrial hot spring (Baquero *et al*. 2020; Liu *et al*. 2021b), providing evidence that EVs are not a laboratory artefact.

It has been suggested that *Sulfolobus* EVs also promote biomineralization (Kish *et al*. 2016). Whilst S-layers have long been implicated in mineral formation, the underlying mechanisms remained unresolved. A study using *Sulfolobus acidocaldarius*, a hyperthermophilic archaeon isolated from metal-enriched environments, demonstrated a passive process of iron phosphate nucleation and growth within the S-layer of cells and cell-free S-layer ‘ghosts’ during incubation in a Fe-rich medium. In addition, EVs of ∼175 nm in diameter were formed and released in response to S-layer encrustation by minerals. These EVs were fully encrusted by minerals, even when cells were only partially encrusted (Kish *et al*. 2016). The authors proposed that these EVs are produced in an attempt to remove sections of damaged S-layer.

Besides *Sulfolobales*, the production of EV vesicles in Crenarchaeota was reported in *I. hospitalis*, a member of the order Desulfurococcales. *Ignicoccus hospitalis* is a diderm archaeon with an inner cytoplasmic membrane and an outer membrane separated by a fairly large periplasmic space (20–1000 nm in width). Vesicles are produced by budding from the inner membrane and numerous vesicles can accumulate in the periplasm and fuse with the outer membrane (Näther and Rachel 2004). *Ignicoccus hospitalis* often hosts cells of the tiny archaeon *N. equitans* attached to its surface (Huber *et al*. 2002; Küper *et al*. 2010). *Nanoarchaeum equitans* has the smallest known genome for an archaeon (0.49 Mb) and cannot synthesize many essential components, including lipids (Waters *et al*. 2003). It is assumed that these components could be delivered from the cytoplasm of *I. hospitalis* to *N. equitans* via vesicles that reach the outer membrane at the position of the symbiont attachment (Junglas *et al*. 2008). Unfortunately, there are presently no genetic tools available to address the mechanism of vesicle production in this fascinating system.

## PRODUCTION OF EVS BY ARCHAEA OF THE PHYLUM EURYARCHAEOTA

**Figure 2.**
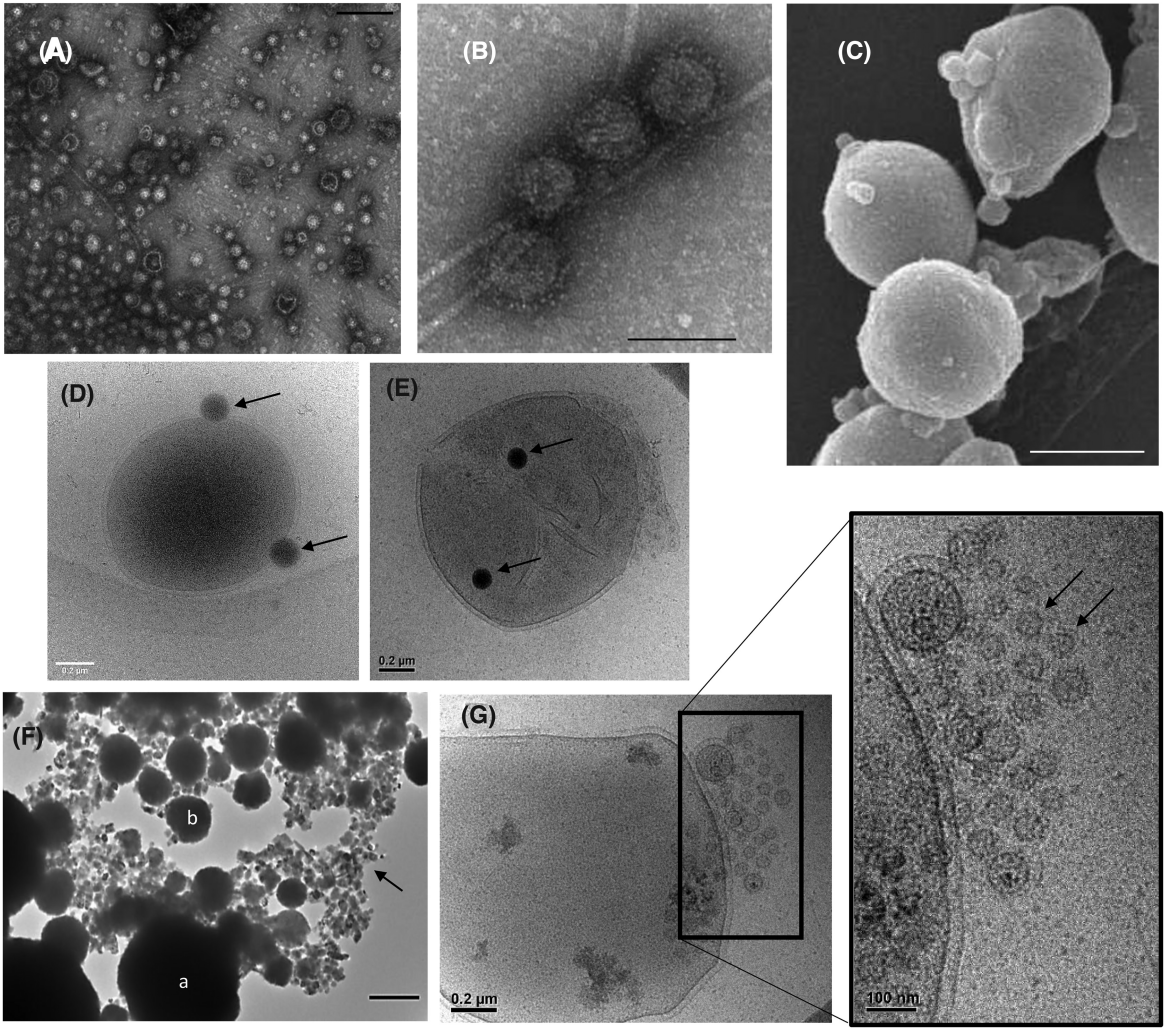
Extracellular vesicles produced by Thermococcales. Transmission electron microscopy images of purified EVs produced by **(A)***T. nautili* showing the size diversity (scale bar, 200 nm) and **(B)** by *T. gammatolerans* (scale bar, 100 nm). **(C)** Scanning electron micrograph of *T. kodakarensis* cells producing EVs (scale bar, 500 nm). Cryo-electron micrographs of **(D)***Thermococcus* sp. 15-2 and **(E)***T. prieurii* cells associated with dark vesicles named sulfur vesicles (SVs). The arrows indicate SVs. **(F)** Transmission electron microscopy image of *T. kodakarensis* cells (a) and EVs (b) entirely mineralized within FeS_2_ pyrite. The EVs are dispersed among Fe_3_S_4_ greigite nanocrystals (indicated by arrow). Scale bar, 500 nm. **(G)** Cryo-electron micrographs of a *T. prieurii* cell producing very small EVs (indicated by an arrow) grouped into a spherical structure at the cell surface.

Most studies dealing with the production of EVs in Euryarchaeota have been performed with archaea of the genus *Thermococcus* (order Thermococcales). Members of the Thermococcales are strictly anaerobic, hyperthermophilic sulfur reducers, which are placed at the base of the Euryarchaeota in most archaeal phylogenies (Adam *et al*. 2017; Da Cunha *et al*. 2017). They are rather easy to cultivate under laboratory conditions, requiring typical equipment for the cultivation of anaerobes, and are typically abundantly present in environmental samples from hydrothermal vents, both terrestrial and marine. Massive production of EVs was first observed in the course of screening for viruses a collection of Thermococcales isolated from hydrothermal deep-sea vents (Soler *et al*. 2008). Subsequently, EVs were also observed in several reference strains widely used as model organisms for the study of hyperthermophiles, such as *Thermococcus kodakarensis* (Gaudin *et al*. 2013; Marguet *et al*. 2013). These EVs (50–150 nm) are covered with the S-layer and are apparently produced by budding (Fig. 2A–C).

Since the genomes of Thermococcales and of other Euryarchaeota do not encode for ESCRT-III homologues (Makarova *et al*. 2010), the mechanism of EV production in this phylum is likely to be different from that postulated for Sulfolobales (Liu *et al*. 2021b). Indeed, the genomes of Thermococcales encode three putative homologues of the Vps4 ATPase, but disruption of any of their genes in *Thermococcus kodakarensis* did not affect EV production (Gill, Catchpole and Forterre, unpublished result). In contrast with the result obtained with *Sulfolobales*, the biochemical characterization of purified EVs from three species of Thermococcales (*T. kodakarensis*,*T. gammatolerans* and *Thermococcus* sp. 5-4) revealed that protein and lipid profiles of EVs and cell membranes from the same species have a similar composition (Gaudin *et al*. 2013). However, the major protein present in both cell membranes and EVs of *Thermococcus* species, the oligopeptide binding protein OppA, was also found in *Sulfolobus* EVs (Ellen *et al*. 2009; Liu *et al*. 2021b).

In addition to the typical EVs, some members of the Thermococcales, such as *T. prieurii* or *T. kodakarensis*, produce numerous intracellular dark vesicles that bud from the host cells (Gorlas *et al*. 2015) (Fig. 2E and F). Energy-dispersive X-ray spectroscopy analyses revealed that these dark vesicles are filled with sulfur, and hence they have been termed ‘sulfur vesicles’ (SVs). The presence of SVs was exclusively observed when elemental sulfur S)0) is added into the growth medium, suggesting that these SVs could be produced to prevent the toxic intracellular accumulation of S(0) and/or polysulfides, thus playing a key role in sulfur detoxification. Surprisingly, the SVs are not produced by all species of Thermococales, suggesting significant differences in the sulfur metabolic pathways (Gorlas *et al*. 2015). More recently, it has been observed that Thermococcales SVs and EVs are actively involved in the production of iron-sulfide biominerals (Gorlas *et al*. 2018), suggesting a defensive function of EVs that might allow Thermococcales to survive in a broad range of extreme environments characterized by the high iron and sulfide contents (Gorlas *et al*. 2018) (Fig. 2F and G).

The EVs produced by members of the Thermococcales were shown to be often associated with either chromosomal or plasmid/viral DNA (Soler *et al*. 2008, 2011; Gaudin *et al*. 2013, 2014; Choi *et al*. 2015). As recently observed with *Sulfolobus* EVs, DNA enclosed within EVs produced by Thermococcales is more resistant to thermodenaturation than free DNA, suggesting a protective role of the EVs (Soler *et al*. 2008), which is likely to be vital for horizontal gene transfer in extreme geothermal environments. The EVs of Thermococcales can indeed transfer DNA between cells. It was demonstrated that EVs of *T. kodakarensis* can be used to transfer plasmid DNA into plasmid-free cells (Gaudin *et al*. 2013).

*Thermococcus onnurineus* cells produce heterogeneous populations of EVs, which differ in terms of size and DNA content (Choi *et al*. 2015). EVs always encapsidate ∼14-kb-long DNA fragments. However, sequencing of the packaged DNA revealed that all regions of the *T. onnurineus* genome are represented in EVs, except for a 9.4-kb region. The authors speculated that this region might participate in DNA packaging and/or EV production (Choi *et al*. 2015). Interestingly, a *T. onnurineus* mutant in which the 9.4-kb region has been deleted still produces EVs but without associated DNA, supporting the original hypothesis (Kim, pers. comm.). This 9.4-kb region encodes various enzymes involved in sulfur metabolism and/or hydrogen production, with the possible roles of these enzymes in DNA packaging being unclear.

Remarkably, EVs produced by *T. nautili*, which contains three plasmids, selectively incorporate only two of these plasmids, pTN1 and pTN3, but not pTN2 (Soler *et al*. 2011; Gaudin *et al*. 2014). The reason for this specificity is unknown and purified EVs did not contain proteins encoded by pTN1 or pTN3. Notably, pTN3 is a defective virus belonging to the viral realm *Varidnaviria* (formerly the PRD1-Adenovirus lineage). This observation reinforces the idea that EVs of Thermococcales can serve as vehicles for the intercellular transport of extrachromosomal DNA (Soler *et al*. 2011; Gaudin *et al*. 2014). EVs containing viral DNA have also been detected in Bacteria and Eukarya (Gill, Catchpole and Forterre 2019) and the term ‘viral vesicle’ has been proposed for these biological entities. Interestingly, *in silico* analysis of the DNA associated with bacterial EVs isolated in diverse marine environments has revealed the presence of many viral genes, suggesting that such ‘viral vesicles’ are abundant in nature, alongside true virions and EVs containing cellular DNA (Soler *et al*. 2015).

Some EVs have been recently detected in cultures of *Methanocaldococcusfervens*, a methanogenic hyperthermophile belonging to the order Methanococcales (Thiroux *et al*. 2021). The natural isolate of *M. fervens* is a lysogen producing a head-tailed virus MFTV1 (Krupovic, Forterre and Bamford 2010; Thiroux *et al*. 2021). *Methanocaldococcusfervens* cultures exposed to copper displayed greater production of EVs, but lower virus production, suggesting an interplay between EV production and virus life cycle (Thiroux *et al*. 2021). Notably, *Methanocaldococcus* and *Thermococcus* species inhabit the same deep-sea hydrothermal vent ecosystems and were shown to share several groups of non-conjugative plasmids, some of which could have been exchanged horizontally through EVs (Krupovic *et al*. 2013). EVs and structures resembling nanotubes were observed in cultures of *Aciduliprofundum boonei* (Reysenbach *et al*. 2006; Reysenbach and Flores 2008), a thermoacidophilic euryarchaeon distantly related to Thermococcales and Methanococcales.

Haloarchaea appear to represent a very promising model for the study of EVs in Archaea. Haloarchaea are halophilic and aerobic microorganisms that thrive in up to 5.5 M NaCl, i.e. salt concentrations approaching saturation, and are responsible for the pink colour of many hypersaline seas and lakes around the globe due to the specific carotenoid pigments that they produce. A seminal study describing EVs produced by *Halorubrum* has uncovered entities blurring the canonical frontiers between plasmids and viruses (Erdmann *et al*. 2017). Indeed, in contrast to plasmid vesicles produced by *T. nautili*, the membranes of EVs from *Halorubrum* carrying the plasmid pR1SE contain mostly proteins encoded by this plasmid, resembling the packaging of viral genomes by capsid proteins. Many of these plasmid-encoded proteins were found in EVs by mass spectrometry analysis. These peculiar ‘plasmid vesicles’ were proposed to be the prototype of a new type of biological entities called ‘plasmidions,’ standing for membrane vesicles mimicking virions (Forterre, Da Cunha and Catchpole 2017). Similar to EVs of *Sulfolobus* (Liu *et al*. 2021b) and *Thermococcus* (Soler *et al*. 2008; Gaudin *et al*. 2014), *Halorubrum* EVs can mediate the transfer of plasmid DNA (pR1SE and derivatives). Notably, the plasmids carried by *Halorubrum* EVs can integrate into haloarchaeal replicons (note that Haloarchaea harbour several circular replicons), and subsequent excision from these replicons generates plasmid derivatives with different segments of the host chromosome, possibly leading to the horizontal transfer of the host genes by vesiduction (Erdmann *et al*. 2017).

## NANOTUBES IN ARCHAEA

It has been known for a long time that eukaryotic cells can produce long tubular structures, often known as tunnelling nanotubes or sometimes microvillus (Lou *et al*. 2012; Rustom 2016; Nawaz and Fatima 2017; Cordero Cervantes and Zurzolo 2021). These nanotubes, formed by extrusion from the cytoplasmic membrane, can have very different lengths and thickness and can contain actin and/or tubulin filaments (for review, see Gill, Catchpole and Forterre 2019). They can connect different cells across substantial distances and have different physiological roles. Nanotubes (also called nanopods) were reported more recently in Bacteria (Baidya *et al*. 2018). It has been proposed that they could play a role in transferring nutrients, electrons or genetic material between cells. Bacterial nanotubes have been extensively studied in *Bacillus subtilis* (Baidya, Rosenshine and Ben-Yehuda 2020 and references therein). More recently, it has been suggested that these structures are not physiologically relevant since ‘they are exclusively extruded from dying cells as a result of biophysical forces’ (Pospíšil *et al*. 2020). The production of nanotubes by bacteria, especially Gram-positive, indeed raises questions because extrusion of nanotubes cannot take place without formation of apertures in the thick peptidoglycan layer. Considering the high number of studies that have emphasized important roles for nanotubes in bacteria during these last few years, the question of their physiological relevance should become a hot topic.

**Figure 3.**
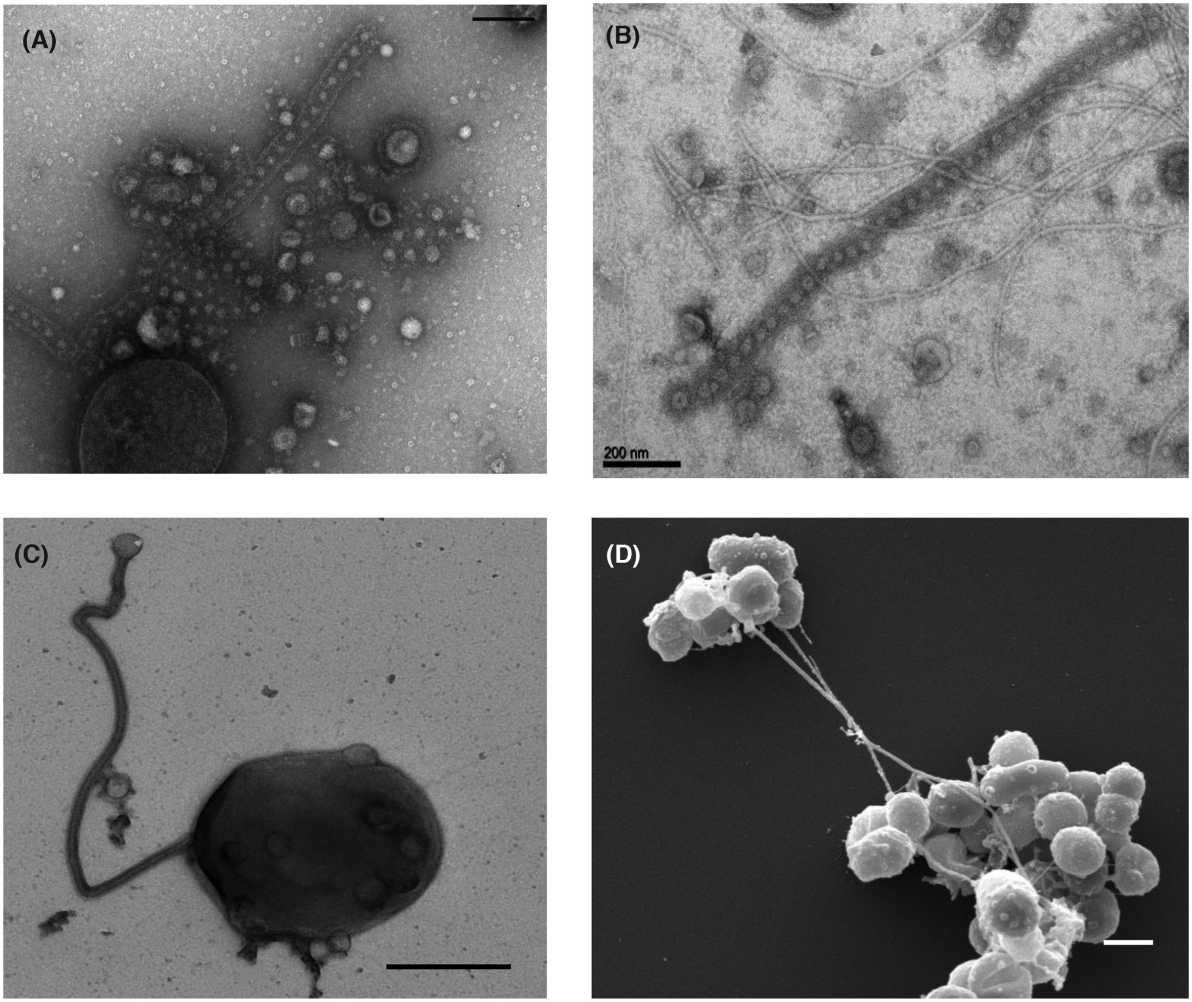
Nanotubes produced by Thermococcales.**(A–C)**Transmission electron microscopy images: **(A)***T. prieurii* cell producing both EVs and nanotubes containing EVs (scale bar, 200 nm); discrete EVs are surrounded by the cellular S-layer forming the nanotube structure. **(B)** Long nanotubes containing EVs produced by *T. prieurii* (scale bar, 200 nm). **(C)** A cell of *Thermococcus* sp. 15-2 producing a long nanotube. **(D)** Scanning electron microscopy image of long nanotubes connecting clusters of *Thermococcus* sp. 15-2 cells. EVs can be also observed at the surface of most cells (scale bar, 1 µm) (Marguet and Forterre, unpublished observations).

In their first studies on the Thermococcales EVs, Forterre and colleagues noticed the presence of strings of EVs enclosed within an elongated membrane structure covered by the S-layer (Soler *et al*. 2008), resembling the nanopods or nanotubes later observed in bacteria (Fig. 3). It remains to be definitively demonstrated that such structures are normally produced by living cells in natural habitats, as in the case of EVs from *Sulfolobales*. However, nanotubes from Thermococcales are sometimes produced in abundance (Soler *et al*. 2008) and throughout different growth phases (Gauliard, pers. comm.), suggesting that they could be physiologically relevant. Some species of Thermococcales are able to produce giant nanotubes that can reach several micrometers in length and are often filled with EVs (Fig. 3). EVs present within these nanotubes are usually smaller than free EVs (Fig. 3A and B) and larger vesicle-like structure are sometimes located in the extremities of the nanotubes (Fig. 3C), suggesting that these structures could be involved in the transport and/or formation of EVs. Nanotubes often connect cells of Thermococcales together (Fig. 3D) and it was suggested that they could be involved in transfer of materials (nucleic acids and proteins) between cells (Marguet *et al*. 2013).

**Figure 4.**
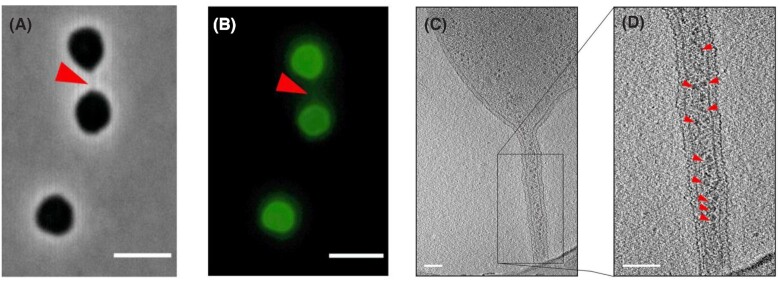
Nanotubes produced by *Haloferax volcanii*. Cells of *H. volcanii* are linked by nanotubes as observed by **(A)** phase-contrast and **(B)** fluorescence microscopy. In panel (B), *H. volcanii* cells are labeled with Alexa Fluor 488. The cell–cell bridge is indicated by a red arrow. Scale bar, 4 µm. **(C, D)** Electron cryo-tomography images of *H. volcanii* nanotubes. In panel (D), ribosomes are indicated by small red arrows. Scale bar, 100 nm. All images are reproduced from Sivabalasarma *et al*. (2020).

During the 80s, the Mevarech group has described an original mechanism of gene transfer between species of the genus *Haloferax* (order Halobacteriales) (Mevarech and Werczberger 1985; Rosenshine, Tchelet and Mevarech 1989). These transfers can be interspecific and were shown to be bidirectional, leading to genetic hybrids through DNA recombination between the parental genomes (Naor *et al*. 2012). They do not seem to involve EVs but instead cell–cell bridges were recently observed by electron cryo-tomography (Sivabalasarma *et al*. 2020) (Fig. 4). The S-layer-covered nanotube-like protrusions are 100 nm in diameter and connect the cells that are up to 2 µm apart (Rosenshine, Tchelet and Mevarech 1989; Sivabalasarma *et al*. 2020) (Fig. 4C). These structures were shown to allow the diffusion of cellular materials, such as ribosomes (Fig. 4D).

A hyperthermophilic crenarchaeon of the genus *Pyrodictium* was found to form extracellular tubules with an outer diameter of around 25 nm, which interconnected the cells and led to the formation of extensive networks (Horn *et al*. 1999; Nickell *et al*. 2003). These extracellular tubes formed by *Pyrodictium* are much thinner than those produced by *Thermococcus* spp. (Soler *et al*. 2008; Marguet *et al*. 2013), and it is not known whether their biogenesis is mechanistically related to the EV production.

Finally, very long tubular structures similar to those observed in Thermococcales have been occasionally observed to be associated with cells of the first cultivated Asgard archaeon, *Prometheoarchaeum synthrophicum* (Imachi *et al*. 2020). However, these nanotubes do not connect cells together, suggesting divergence in physiological role. Imachi and co-workers have suggested that similar structures present in the Asgard ancestor of eukaryotes have facilitated the engulfment of an aerobic bacterial symbiont at the onset of eukaryogenesis (Imachi *et al*. 2020).

## PERSPECTIVES

Although the research on archaeal EVs and nanotubes is still in its infancy, it is already clear that these structures, especially EVs, play a profound role in archaeal physiology and environmental adaptation. Archaeal EVs were shown to contain cellular, plasmid or viral DNA. Vesiduction has now been demonstrated for several archaeal phyla (Thermococcales, Haloarchaea and Sulfolobales). As a result, archaeal EVs probably play an important role in the evolution and plasticity of cellular archaeal genomes and their mobilome, thus enabling archaeal adaptation in very diverse and often extreme environments. Future investigations on archaeal EVs should now more systematically test their ability to facilitate horizontal gene transfers by vesiduction in order to have a more complete overview of their importance in archaeal evolution. Moreover, the molecular mechanism of DNA recruitment into EVs is yet to be understood.

The existence of diverse mechanisms of EV production in Archaea is exemplified by the abundant production of EVs in Crenarchaeota and Euryarchaeota that encode and lack the ESCRT system, respectively. The discovery of proteins involved in EV production in Thermococcales and Haloarchaea is a major challenge right now. Hopefully, the availability of powerful genetic tools for these organisms will help to identify such proteins in the near future. One possibility is that some proteins involved in cell division in Euryarchaeota are also involved in EV production, as is the case for ESCRT proteins in Crenarchaeota. Another interesting possibility is that EV formation depends on proteins involved in polar lipids biosynthesis, especially those involved in the modification of the polar head groups. It was suggested that EV production in Bacteria and Eukarya is controlled, at least partly, by the regulation of enzymes involved in the incorporation of polar lipids in order to create asymmetry between the outer and inner membrane leaflets (Gill, Catchpole and Forterre 2019). However, this hypothesis cannot be directly transposed to Archaea that often have monolayer membranes formed by long tetraether lipids. In that case, the classical models of membrane fission and fusion that involve the transient opening of the bilayer cannot be applied (Relini *et al*. 1996). Notably, monolayer membranes are especially prevalent in Sulfolobales and Thermococcales, which have been more thoroughly studied for EV production. Indeed, in many Sulfolobales species, 95–100% of lipids in the membrane are membrane-spanning C_40_ glycerol dibiphytanyl glycerol tetraethers (Quemin *et al*. 2015; Kasson *et al*. 2017; Liu *et al*. 2018; Wang *et al*. 2019). One possibility is that membrane curvature in organisms with monolayer membranes is induced by the accumulation of larger polar head groups in the outer surface of the monolayer. Alternatively, patches of membrane could adopt bilayer structure by favouring the local clustering of diether lipids or that of tetraether lipids adopting the horseshoe conformation, as recently observed in the membranes of some archaeal viruses (Kasson *et al*. 2017). In the future, a better knowledge of the archaeal lipid biosynthetic pathways, especially that of the formation of polar head group, would enable the genetic manipulations necessary to test different hypotheses.

The production of nanotubes observed in some Archaea is another aspect worthy of further studies. Curiously, although nanotubes have not yet been observed in Sulfolobales, some species of Thermococcales produce abundant nanotubes that could be linked to EV production. Studying the still mysterious mechanisms for EV production in these archaea thus should also bring information on nanotubes, their relevance and physiological roles. Interestingly, it was shown that some nanotubes in eukaryotes are formed from EVs (Rustom 2016). This suggests that nanotubes could be also relevant structure in Archaea and studying their connection to EVs is likely to become a hot topic of research in the near future. It will be especially interesting to see whether, besides Lokiarchaeota, other Asgard archaea produce nanotubes and whether the eukaryotic-like actins encoded by these archaea are involved in the formation of these structures. A possible evolutionary connection between the pathway for nanotube formation in Archaea and Eukaryotes will be worth exploring in Asgard archaea or Bathyarchaea, which encode actin and/or tubulin homologues.

## ACKNOWLEDGEMENTS

The authors would like to thank Emilie Gauliard and Evelyne Marguet for electron micrographs of Fig. 3C and D.

## FUNDING

The work of extracellular vesicles and nanotubes was supported in our laboratories by a European Research Council grant from the European Union Seventh Framework Programme (FP/2007–2013)/Project EVOMOBIL-ERC Grant Agreement 340440 to PF, and Agence Nationale de la Recherche (#ANR-17-CE15-0005-01) and Ville de Paris Emergence(s) program (project MEMREMA) grants to MK. JL was partly supported through the PRESTIGE post-doctoral program from European Union Seventh Framework Programme. AG was supported by the Agence Nationale de la Recherche, project HYPERBIOMIN (ANR-20-CE02-0001-01).
